# Update on Sedation for Upper Gastrointestinal Endoscopy in Japan: Current Evidence on Remimazolam

**DOI:** 10.1002/deo2.70402

**Published:** 2026-08-12

**Authors:** Ryoji Ichijima, Masahiro Toyama, Taiki Shishido, Yuki Koyo, Takehiro Yoshii, Reona Kawamura, Tsubasa Ishikawa, Yasuhiko Mizuguchi, Naoya Toyoshima, Hiroyuki Takamaru, Masayoshi Yamada, Masau Sekiguchi, Seiichiro Abe, Yutaka Saito

**Affiliations:** ^1^ Endoscopy Division National Cancer Center Hospital Tokyo Japan; ^2^ Division of Screening Technology National Cancer Center Institute for Cancer Control Tokyo Japan

**Keywords:** elderly, recovery, remimazolam, sedation, upper gastrointestinal endoscopy

## Abstract

Sedation is an essential component of upper gastrointestinal endoscopy because it reduces patient discomfort and anxiety while improving patient acceptance and procedural quality. In Japan, benzodiazepines such as midazolam and opioid analgesics have long been widely used in routine clinical practice; however, many of these agents historically lacked formal insurance approval for sedation during gastrointestinal endoscopy. Propofol is also a useful sedative because of its rapid onset and recovery, but its use in Japan has been limited because of concerns regarding respiratory and cardiovascular depression and the need for management by anesthesia‐trained personnel. Against this background, the approval of remimazolam for sedation during gastrointestinal endoscopy in June 2025 represented a major turning point in Japanese endoscopic sedation practice. Remimazolam is an ultra‐short‐acting benzodiazepine rapidly metabolized by carboxylesterases and reversible with flumazenil. Japanese phase III trials demonstrated high sedation success rates and rapid recovery during upper gastrointestinal endoscopy, including in elderly patients. Compared with midazolam, remimazolam shortened awakening and ambulation times, whereas compared with propofol, it may cause fewer respiratory and cardiovascular adverse events. This review summarizes the current status of upper gastrointestinal endoscopic sedation in Japan, focusing on the clinical evidence and positioning of the newly approved ultra‐short‐acting benzodiazepine, remimazolam.

## 1 Introduction

Upper gastrointestinal endoscopy is indispensable for the diagnosis and treatment of not only malignant tumors such as gastric cancer, esophageal cancer, and duodenal tumors, but also peptic ulcer disease, infectious disorders, and functional gastrointestinal diseases. In recent years, advances in diagnostic technologies such as image‐enhanced endoscopy and magnifying endoscopy, together with the development of therapeutic procedures including endoscopic submucosal dissection (ESD), have further increased the level of diagnostic and therapeutic accuracy required in upper gastrointestinal endoscopy. However, gag reflexes, nausea, anxiety, pain, and prolonged procedural duration impose a considerable burden on patients and may lead to procedural intolerance and body movement during treatment. Appropriate sedation is therefore important not only for reducing patient discomfort but also for maintaining a stable endoscopic field and endoscopic maneuverability.

The demand for sedation during upper gastrointestinal endoscopy has increased in Japan. Elderly patients frequently undergo upper gastrointestinal endoscopy for screening, diagnosis, and treatment because the incidence of gastrointestinal malignancies is higher in this population. On the other hand, elderly patients have reduced cardiopulmonary reserve, impaired hepatic and renal function, cognitive dysfunction, swallowing impairment, and reduced muscle strength, making them more vulnerable to hypoxemia, hypotension, delayed recovery, falls, and aspiration associated with sedative administration [1]. Therefore, sedation strategies in Japanese upper gastrointestinal endoscopy must achieve an appropriate balance among adequate sedation, rapid recovery, and safety. In Japan, many outpatient endoscopic procedures are performed without anesthesiologist involvement because of limited medical resources and workforce imbalance. Before June 2025, few sedatives had formal insurance approval for gastrointestinal endoscopic sedation in Japan. Traditionally, benzodiazepines centered on midazolam have been widely used in Japanese endoscopic practice. However, for many years, these agents lacked formal approval for sedation during gastrointestinal endoscopy. Propofol is characterized by rapid induction and recovery, but because of its substantial effects on respiratory and hemodynamic stability, its administration generally requires supervision by physicians experienced in anesthesia management. Consequently, institutional and personnel limitations have restricted its widespread use in routine endoscopic practice in Japan. Against this background, the approval of remimazolam for sedation during gastrointestinal endoscopy in Japan in June 2025 represented an important milestone in Japanese endoscopic sedation practice. In this review, we summarize the current status of sedation in Japan and discuss the clinical role of remimazolam, focusing on diagnostic and therapeutic upper gastrointestinal endoscopy.

## 2 Current Status of Sedation for Upper Gastrointestinal Endoscopy in Japan

Sedation practice in Japan has evolved under healthcare systems, drug approval processes, human resources, and clinical cultures distinct from those in Western countries. In Europe and North America, deep sedation using propofol with the involvement of anesthesiologists or anesthesia‐trained personnel is relatively common in some institutions. In contrast, sedation in Japan is frequently managed by endoscopists and endoscopy unit staff, and sedative selection is strongly influenced by safety and practical operability.

Traditionally, benzodiazepines such as midazolam, diazepam, and flunitrazepam have been widely used during upper gastrointestinal endoscopy in Japan [1]. These agents provide anxiolytic and amnestic effects that are highly compatible with endoscopic procedures. However, because of their relatively long half‐lives, delayed awakening and prolonged unsteadiness may occur, especially in elderly patients and those with impaired hepatic function. Furthermore, concomitant use of opioid analgesics may increase the risk of respiratory depression.

Propofol provides rapid induction and recovery and facilitates deep sedation. However, it lacks analgesic effects and may cause dose‐dependent respiratory and cardiovascular depression. Therefore, institutions using propofol require systems capable of advanced airway management and resuscitation. In Japan, shortages of anesthesiologists and institutional differences in safety management systems have limited the widespread adoption of propofol for routine upper gastrointestinal endoscopy.

Dexmedetomidine, an alpha‐2 adrenergic receptor agonist, is another sedative option used in Japan, particularly for prolonged therapeutic procedures such as ESD. Its limited respiratory depressive effect is a potential advantage, whereas slow onset, bradycardia, and hypotension remain important limitations. Previous studies and meta‐analyses have suggested the feasibility of dexmedetomidine‐based sedation for gastrointestinal endoscopy and ESD; however, careful hemodynamic monitoring is required [1, 2, 3]

Remimazolam is an ultra‐short‐acting benzodiazepine that acts on the benzodiazepine‐binding site of the gamma‐aminobutyric acid subtype A (GABAA) receptor and enhances the inhibitory effects of GABA. Its most important pharmacological characteristic is rapid hydrolysis by carboxylesterase 1 into inactive metabolites. Remimazolam may also be useful in patients with impaired hepatic function because it is rapidly metabolized by tissue carboxylesterases rather than hepatic cytochrome P450 enzymes. This pharmacokinetic profile may reduce delayed recovery associated with hepatic dysfunction compared with conventional benzodiazepines such as midazolam [4]. Particularly in outpatient upper gastrointestinal endoscopy and elderly patients, where postprocedural ambulation and discharge readiness are clinically important, the pharmacokinetic profile of remimazolam appears highly suitable for Japanese clinical practice.

Table 1 summarizes the characteristics, advantages, and limitations of the major sedatives currently used in upper gastrointestinal endoscopy in Japan.

**TABLE 1:** Simplified comparison of major sedatives used for upper gastrointestinal endoscopy.

**Agent**	**Approximate half‐life/specific antagonist**	**Japanese GI‐endoscopy approval and reimbursement**	**Administration, continuous use, and analgesia**	**Respiratory and circulatory effects**	**Recommended clinical situations**
Midazolam	Elimination half‐life approximately 1.8–6.4 h/flumazenil	No GI‐endoscopy‐specific indication; used off‐label	Slow IV bolus with titrated supplemental boluses; continuous infusion is not standard; no intrinsic analgesia	Dose‐dependent respiratory depression and hypotension may occur	Familiar and inexpensive option for short routine procedures; recovery may be delayed, particularly in older patients or hepatic dysfunction
Propofol	Rapid redistribution; terminal elimination extends for hours/no specific antagonist	No GI‐endoscopy‐specific indication; used off‐label	Repeated IV boluses or continuous infusion; allows fine titration but requires trained personnel; no intrinsic analgesia	Marked dose‐dependent respiratory depression, apnea, hypotension, and bradycardia occur	Reliable deep sedation and rapid clinical recovery when dedicated monitoring, airway rescue, and appropriately trained staff are available
Dexmedetomidine	Elimination half‐life approximately 2 h/no specific antagonist	A broader indication exists for sedation during non‐intubated procedures under local anesthesia	Loading dose followed by continuous infusion; modest opioid‐sparing effect but not sufficient as sole analgesia	Relatively limited respiratory depression; bradycardia, hypotension, and transient hypertension may occur	Potential option for prolonged therapeutic procedures when preservation of spontaneous ventilation is important; careful hemodynamic monitoring is required
Remimazolam	Elimination half‐life approximately 0.75 h/flumazenil	Approved in Japan for sedation during GI endoscopy	Slow IV bolus with titrated supplemental boluses; continuous infusion is not included in the current GI‐endoscopy regimen; no intrinsic analgesia	Generally, show less cardiorespiratory depression than propofol	Useful when rapid recovery and reversibility are priorities, including outpatient, older, or higher‐risk patients; repeated boluses may be needed during prolonged procedures

## 3 Positioning of Remimazolam in International Sedation Guidelines

Although remimazolam has attracted increasing attention as a novel sedative for gastrointestinal endoscopy, its positioning in international sedation guidelines remains evolving. Table 2 summarizes the current positioning of remimazolam in major international sedation guidelines [5, 6, 7]. The American Society for Gastrointestinal Endoscopy guideline on sedation and anesthesia in gastrointestinal endoscopy was published before remimazolam became widely available; therefore, remimazolam is not specifically positioned as a standard sedative in that guideline. In contrast, the British Society of Gastroenterology guideline, which incorporated more recent advances in endoscopic sedation, describes remimazolam as a short‐acting benzodiazepine with a potentially favorable profile, including limited cardiovascular and respiratory depression, reduced injection pain, and reversibility with flumazenil. The guideline also notes that remimazolam can be administered as a single dose, repeated boluses, or infusion for procedural sedation. However, unlike midazolam and propofol, remimazolam has not yet been fully established as a standard sedative in most international guidelines, mainly because clinical experience and real‐world evidence remain limited. Therefore, the role of remimazolam should currently be regarded as promising but still evolving, particularly in comparison with conventional agents such as midazolam and propofol. In Japan, where the use of propofol is often limited by workforce and safety‐management issues, remimazolam may have a particularly important clinical role as a benzodiazepine‐based sedative that combines rapid recovery with reversibility.

**TABLE 2:** Position of remimazolam in International Sedation Guidelines.

**Guideline**	**Position of remimazolam**	**Key comments**
ESGE guideline (2015) [6]	Not specifically addressed	Published before the clinical introduction of remimazolam
ASGE guideline (2018) [5]	Not specifically established	Published before widespread clinical introduction
BSG guideline (2024) [7]	Recognized as a promising short‐acting benzodiazepine	Rapid recovery and reversibility with flumazenil are noted

## 4 Clinical Evidence in Upper Gastrointestinal Endoscopy

Table 3 summarizes the selected clinical studies evaluating the efficacy, recovery, and safety of remimazolam for upper gastrointestinal endoscopy. A Japanese investigator‐initiated phase II dose‐ranging study [8] evaluated the optimal dose of remimazolam for gastrointestinal endoscopic sedation. An initial dose of 3 mg followed by an additional 1 mg bolus administration was considered effective and safe and subsequently became the basis for later phase III trials and the approved Japanese dosing regimen. Because upper gastrointestinal endoscopy is generally a relatively short procedure, the goal of sedation is to suppress discomfort and gag reflexes while avoiding oversedation.

**TABLE 3:** Selected clinical studies of remimazolam relevant to upper gastrointestinal endoscopy.

**Study (year)**	**Country**	**Design and participants**	**Comparator or setting**	**Key findings**
Borkett et al. (2015) [13]	United States	Phase IIa randomized, double‐blind dose‐finding study; diagnostic upper GI endoscopy; *n* = 100	Single‐dose remimazolam 0.10, 0.15, or 0.20 mg/kg versus midazolam 0.075 mg/kg	Procedure success was 32%, 56%, and 64% with the three remimazolam doses versus 44% with midazolam. Remimazolam produced faster sedation onset (1.5–2.5 vs. 5 min) with rapid recovery and a similar safety profile.
Ichijima et al. (2022) [8]	Japan	Single‐center, open‐label, uncontrolled phase II dose‐ranging study; *n* = 40 (20 upper GI endoscopies and 20 colonoscopies)	Initial/additional doses of 2/1 mg and 3/1 mg; no active comparator	Sedation was successful in all procedures in the evaluated cohorts. The 3‐mg initial and 1‐mg supplemental regimen was selected; no patient required flumazenil or manual ventilation.
Lu et al. (2022) [15]	China	Multicenter randomized controlled trial; patients aged 65–85 years undergoing diagnostic upper GI endoscopy; *n* = 400	Remimazolam versus propofol; both combined with fentanyl	Hypotension (36.5% vs. 69.6%), bradycardia (1.5% vs. 8.5%), and respiratory depression (4.5% vs. 10.0%) were less frequent with remimazolam; sedation efficacy was clinically comparable.
Ichijima et al. (2024) [9]	Japan	Multicenter, randomized, double‐blind, placebo‐controlled phase III trial; diagnostic upper GI endoscopy; *n* = 48	Remimazolam (*n* = 37) versus placebo (*n* = 11)	Sedation success was 91.9% versus 9.1% (*p* < 0.01). Recovery was rapid, and no severe adverse events were reported.
Ichijima et al. (2025) [10]	Japan	Age‐stratified secondary analysis; upper GI endoscopy; *n* = 56 (45 non‐elderly and 11 elderly patients)	Elderly (at least 65 years) versus non‐elderly remimazolam recipients	Sedation success was 100% versus 95.6%. Elderly patients required a lower total dose (3.0 vs. 4.0 mg), although time to regain walking ability was longer; no severe adverse events occurred.
Ichijima et al. (2025) [11]	Japan	Retrospective propensity score‐matched study; diagnostic upper GI endoscopy; 34 matched patients per group	Remimazolam versus midazolam	Median time to awakening was 0 versus 27 min, and median time to discharge readiness was 5 versus 39 min (both *p* < 0.01). Adverse events were infrequent.
Ikehara et al. (2025) [17]	Japan	Prospective single‐arm phase III trial of therapeutic endoscopic procedures; *n* = 62	Remimazolam plus opioid analgesics; no active comparator	Sedation success was 93.5%, and preprocedural sedation success was 98.4%. Median times to adequate sedation and discharge readiness were 4 and 2 min; no severe adverse events occurred.
Yamaguchi et al. (2026) [12]	Japan	Prospective, multicenter, randomized, single‐blind trial; diagnostic upper GI endoscopy; *n* = 38	Remimazolam (*n* = 20) versus midazolam (*n* = 18)	Independent ambulation at 5 min was achieved by 85.0% versus 0%. Mean time to walking was 4.25 versus 35.56 min, and hypoxemia occurred in 5.0% versus 33.3%.
Park et al. (2026) [14]	South Korea	Seven‐center, randomized, single‐blind phase III trial; diagnostic upper GI endoscopy; *n* = 132	Remimazolam (*n* = 66) versus midazolam (*n* = 66)	Total procedure time was 30.3 versus 48.5 min (*p* < 0.001), with shorter induction, recovery, and discharge times. Overall adverse‐event rates were similar, and patient satisfaction was higher with remimazolam.

Abbreviations: ESD, endoscopic submucosal dissection; GI, gastrointestinal.

A Japanese phase III placebo‐controlled trial evaluated the efficacy and safety of remimazolam in patients undergoing upper gastrointestinal endoscopy [9]. The primary endpoint, sedation success, was defined as a composite outcome consisting of achievement of a Modified Observer's Assessment of Alertness/Sedation (MOAA/S) score ≤4 before endoscope insertion, completion of the procedure, and maintenance of sedation within a predefined rescue dosing limit.

The sedation success rate was 91.9% (34/37) in the remimazolam group compared with 9.1% (1/11) in the placebo group (*p* < 0.001). In addition, 91.9% of patients achieved adequate sedation before endoscope insertion. Recovery was also rapid. The median time from the end of endoscopy to awakening was 0.0 min, whereas the median time to regain ambulation was 5.0 min. In short diagnostic upper gastrointestinal endoscopy procedures, these characteristics may shorten recovery room stay, improve outpatient flow, and enhance endoscopy unit efficiency. Particularly in Japan, where many upper gastrointestinal endoscopic procedures are performed in outpatient settings, early and safe discharge after endoscopy is clinically important.

Regarding safety, hypoxemia requiring oxygen administration occurred in a small number of patients, but only a few required emergency flumazenil administration or airway intervention. These findings suggest that remimazolam provides an acceptable safety profile during diagnostic upper gastrointestinal endoscopy when appropriate monitoring and rescue systems are available. Nevertheless, hypoxemia cannot be completely avoided, and continuous monitoring of oxygen saturation, blood pressure, and consciousness level, together with preparation for oxygen administration and airway management, remains essential.

## 5 Efficacy and Safety in Elderly Patients

Sedation in elderly patients is one of the most important issues in upper gastrointestinal endoscopy. Japan is a rapidly aging society, and elderly patients frequently undergo upper gastrointestinal endoscopy for screening, diagnosis, and treatment of gastric and esophageal malignancies. Elderly individuals may exhibit increased sensitivity to sedatives, making deep sedation more likely even with standard doses. In addition, comorbidities, polypharmacy, malnutrition, sarcopenia, and frailty may influence the occurrence of sedation‐related adverse events.

An age‐stratified analysis of the REM‐IICT‐JP01 trial demonstrated a sedation success rate of 100% (11/11) in elderly patients and 95.6% (43/45) in non‐elderly patients, indicating favorable sedative efficacy even in elderly individuals. The total dose of remimazolam was significantly lower in elderly patients than in non‐elderly patients (3.0 [2.0–4.0] mg vs. 4.0 [3.0–8.0] mg, *p* < 0.01), suggesting that adequate sedation may be achieved with lower doses in elderly individuals [10].

Although no significant difference in awakening time was observed, the time required to regain walking ability was significantly longer in elderly patients (5.0 [0–60] min vs. 5.0 [0–30] min, *p* = 0.03). Regarding safety, hypotension occurred in two non‐elderly patients (4.4%), whereas hypoxemia occurred in one elderly patient (9.1%). Importantly, no severe adverse events occurred, and no patients required flumazenil administration or bag‐valve‐mask ventilation. These findings suggest that remimazolam may provide effective and relatively safe sedation even in elderly patients when cautious dose titration and careful monitoring are applied.

## 6 Comparison with Midazolam

Midazolam has long been one of the most widely used sedatives in gastrointestinal endoscopy in Japan. Although it provides sedative, anxiolytic, and amnestic effects and is familiar to endoscopists, its relatively long half‐life may result in delayed awakening and prolonged postprocedural observation, particularly in elderly patients and those with impaired hepatic function.

The first Japanese comparative study between remimazolam and midazolam was conducted as a subanalysis of the REM‐IICT‐JP01 trial [11]. A retrospective propensity score‐matched analysis comparing upper gastrointestinal endoscopy performed under remimazolam or midazolam sedation demonstrated that the median time from completion of endoscopy to awakening was significantly shorter in the remimazolam group than in the midazolam group (0 [0–5.0] min vs. 27.0 [23.0–40.5] min). Furthermore, the median time to discharge readiness or ambulation was significantly shorter in the remimazolam group (5.0 [0–5.0] min vs. 39.0 [35.0–52.5] min; both *p* < 0.01).

In addition, the multicenter prospective randomized RECOVER study [12] comparing remimazolam and midazolam in sedated upper gastrointestinal endoscopy demonstrated that 85.0% (17/20) of patients in the remimazolam group were able to walk independently 5 min after endoscopy, whereas none of the patients in the midazolam group achieved this endpoint (0/18, *p* < 0.0001). The mean time to ambulation was significantly shorter with remimazolam (4.25 min vs. 35.56 min). Awakening time and discharge readiness were also significantly improved in the remimazolam group.

These differences are clinically important in outpatient upper gastrointestinal endoscopy. Delayed recovery prolongs recovery room occupancy, increases nursing burden, and delays patient discharge. The rapid recovery associated with remimazolam may therefore improve not only patient satisfaction but also overall endoscopy unit workflow efficiency.

Consistent with Japanese studies, international clinical trials have also demonstrated the recovery advantages of remimazolam during diagnostic upper gastrointestinal endoscopy. In a phase IIa randomized double‐blind study, Borkett et al. reported that remimazolam achieved effective procedural sedation with a more rapid recovery profile than midazolam [13]. More recently, a multicenter randomized controlled trial conducted in Korea demonstrated significantly shorter induction, recovery, and discharge times with remimazolam compared with midazolam while maintaining a comparable safety profile [14]. These findings suggest that the recovery benefits of remimazolam are reproducible across different healthcare systems and clinical settings.

## 7 Comparison with Propofol

Propofol is widely used worldwide because of its rapid onset and reliable deep sedation. Particularly in short procedures, it provides excellent patient comfort and procedural efficiency. However, propofol has a relatively narrow therapeutic window, and even small dose increases may induce deep sedation or conditions close to general anesthesia. Respiratory depression, hypotension, and injection pain are well‐recognized adverse events.

Although few studies directly comparing remimazolam and propofol have been conducted in Japan because the number of institutions routinely using propofol remains limited, international studies suggest that sedation success rates are generally comparable between the two agents [15]. Meta‐analyses have reported that remimazolam may reduce the incidence of hypotension and hypoxemia compared with propofol [16]. These characteristics may be particularly advantageous in elderly patients and those with impaired cardiopulmonary reserve.

On the other hand, propofol enables rapid induction and stable deep sedation. With remimazolam, additional bolus administration may occasionally be required before reaching the target sedation depth, resulting in slightly slower induction. Furthermore, excessive repeated bolus administration without sufficient assessment intervals may lead to delayed oversedation. Therefore, adherence to appropriate dosing intervals and careful assessment of sedation depth using tools such as the MOAA/S scale are essential.

In Japanese endoscopic practice, sufficient personnel, airway management capabilities, and emergency response systems for safe propofol use are not always available. In institutions where anesthesiologist involvement is limited or outpatient procedures predominate, remimazolam may offer practical advantages because of its reversibility with flumazenil. Therefore, sedative selection should be based not only on recovery time but also on patient background, procedural characteristics, institutional resources, and safety management systems.

## 8 Sedation for Upper Gastrointestinal ESD and the Role of Remimazolam

Upper gastrointestinal ESD, including gastric and esophageal ESD, is a minimally invasive treatment enabling en bloc resection of early malignant neoplasms. Compared with diagnostic upper gastrointestinal endoscopy, ESD procedures are substantially longer and involve stronger procedural stimulation associated with mucosal incision, submucosal dissection, and hemostatic interventions. Consequently, patient movement, coughing, and nausea may directly affect procedural safety and therapeutic precision. An ideal sedative for ESD should therefore provide sufficient sedation depth, stable respiratory and cardiovascular dynamics, and rapid postprocedural recovery.

Remimazolam appears promising for sedation during ESD. In the Japanese multicenter prospective REM‐IICT‐JP02 trial [17], the efficacy and safety of remimazolam combined with opioid analgesics for therapeutic endoscopic procedures were evaluated. ESD accounted for 62.3% of all enrolled procedures. The sedation success rate was 93.5%, whereas successful preprocedural sedation was achieved in 98.4% of patients. The median time from initial administration to adequate sedation was 4.0 min, and the median time from procedure completion to discharge readiness was 2.0 min, indicating rapid recovery.

However, the ultra‐short‐acting nature of remimazolam may increase the number of additional bolus administrations during prolonged procedures such as ESD. In the REM‐IICT‐JP02 trial, the mean total number of administrations was 10.7, and longer procedures tended to require more repeated dosing. The investigators suggested that optimization of additional dosing strategies may be necessary during prolonged procedures.

As a practical local approach at our institution, a syringe pump is used to assist repeated small‐volume bolus administration of remimazolam during prolonged upper gastrointestinal ESD. For example, remimazolam can be prepared in a syringe and administered using a pump setting that delivers each additional dose at a controlled speed rather than by rapid manual injection. Importantly, this approach should be regarded as a local practical strategy for repeated bolus administration, not as an established continuous infusion regimen, and its efficacy and safety require prospective validation.

Regarding safety, adverse events occurred in 35.5% of patients, although most were mild or moderate. Hypoxemia (SpO_2_ < 94%) occurred in 12.9% of patients but improved with temporary oxygen administration in all cases. These findings suggest that remimazolam may provide feasible and relatively safe sedation during upper gastrointestinal ESD when appropriate monitoring systems and oxygen administration protocols are available.

An additional advantage of remimazolam is its reversibility with flumazenil. In Japanese clinical practice, where elderly patients and individuals with comorbidities increasingly undergo ESD, the combination of rapid recovery and reversibility may contribute to safer sedation management. Furthermore, compared with propofol, remimazolam may exert less hemodynamic suppression, potentially benefiting elderly patients and those with cardiovascular disease.

Nevertheless, several challenges remain regarding remimazolam use during ESD. First, the current Japanese administration strategy relies primarily on initial and repeated bolus dosing, which may increase the burden on endoscopists and sedation personnel during prolonged procedures. Second, because remimazolam itself lacks analgesic effects, concomitant use of opioid analgesics such as pethidine or fentanyl is necessary, increasing the risk of respiratory depression. Third, precise titration of sedation depth is essential because insufficient sedation may result in body movement, whereas excessive sedation may induce respiratory suppression.

Outside Japan, several combination strategies using adjunctive agents together with remimazolam have been explored for gastrointestinal endoscopic procedures. In addition to opioid analgesics, agents such as esketamine, ketamine, and low‐dose propofol have been evaluated in preliminary studies [18, 19]. Although these approaches may offer potential advantages in selected situations, current evidence remains limited and further studies are required to clarify their clinical role and optimal indications.

Therefore, the optimal administration strategy for remimazolam during ESD should be considered separately from that used in diagnostic upper gastrointestinal endoscopy. Future studies should establish standardized protocols including initial dose, rescue dosing interval, concomitant analgesics, oxygen administration, capnography, flumazenil criteria, and postprocedural observation duration. Prospective comparative studies between remimazolam, propofol, and midazolam during prolonged ESD procedures are warranted.

## 9 Safety Considerations

Although remimazolam is expected to provide relatively favorable safety, sedation‐related adverse events such as hypoxemia, respiratory depression, hypotension, bradycardia, and delayed awakening cannot be completely avoided. In upper gastrointestinal endoscopy, pharyngeal stimulation, secretion retention, airway obstruction, vomiting, and aspiration remain important concerns. Therefore, appropriate monitoring and emergency response systems are as important as sedative selection itself.

Basic monitoring should include regular assessment of consciousness level, oxygen saturation, blood pressure, heart rate, and respiratory status. In high‐risk patients and prolonged procedures, capnography may provide additional information regarding ventilation status. When hypoxemia occurs, oxygen administration, jaw‐lift maneuver, positional adjustment, suctioning, and bag‐valve‐mask ventilation should be immediately available.

Electroencephalographic monitoring such as bispectral index (BIS) may support sedation assessment, although interpretation during benzodiazepine sedation may differ from that during propofol sedation. Consequently, sedation management during remimazolam administration should integrate clinical assessment tools such as the MOAA/S scale together with respiratory and hemodynamic evaluation rather than relying solely on BIS values [20, 21].

## 10 Current Limitations and Future Perspectives

In Japan, the usefulness of remimazolam has also been reported not only in upper gastrointestinal endoscopy but also in colonoscopy, endoscopic ultrasonography, and pancreatobiliary endoscopic procedures such as endoscopic retrograde cholangiopancreatography. Recent Japanese studies demonstrated favorable sedation efficacy, rapid recovery, and acceptable safety profiles in these procedures [22, 23, 24, 25, 26, 27, 28]. Although remimazolam has substantial potential in Japanese endoscopic sedation, several limitations remain.

First, the cost of remimazolam is higher than that of conventional benzodiazepines such as midazolam [1], which may limit widespread adoption.

Second, prolonged procedures such as upper gastrointestinal ESD may require frequent repeated bolus administration, increasing the burden on sedation personnel. Establishment of optimized administration strategies, including continuous infusion protocols, remains an important future challenge.

Third, accumulation of real‐world evidence is essential. Although clinical trials strictly regulate patient background and dosing protocols, actual clinical practice includes elderly patients, obesity, obstructive sleep apnea, dementia, anticoagulant use, and concomitant opioid administration. Prospective registries and multicenter collaborative studies are necessary to comprehensively evaluate rare adverse events, hypoxemia, hypotension, delayed recovery, flumazenil administration, falls, aspiration, and endoscopy unit efficiency.

Sedative responsiveness differs substantially among individuals. In the future, individualized sedation algorithms may become necessary. Integration of artificial intelligence‐based systems capable of evaluating respiratory patterns, body movement, and sedation depth may contribute to the development of safer and more efficient automated sedation management systems for upper gastrointestinal endoscopy.

## 11 Conclusion

The demand for sedation during upper gastrointestinal endoscopy continues to increase. Remimazolam is an ultra‐short‐acting benzodiazepine characterized by rapid recovery, reversibility with flumazenil, and relatively stable respiratory and cardiovascular dynamics. Following its approval for gastrointestinal endoscopic sedation in Japan in 2025, remimazolam has emerged as an important new option in Japanese endoscopic practice.

By integrating Japan's regulatory and staffing context with recent phase II/III and comparative evidence, this review clarifies the clinical settings in which remimazolam may offer practical advantages, including for older adults and therapeutic endoscopy. It also highlights remaining evidence gaps regarding prolonged procedures and high‐risk populations.

Further accumulation of real‐world evidence will be important for addressing these gaps and establishing optimal sedation strategies according to patient background and institutional resources. Remimazolam may play an important role in achieving both safety and procedural efficiency in upper gastrointestinal endoscopic sedation in Japan.

## Author Contributions

**Ryoji Ichijima** drafted the manuscript. **Masahiro Toyama**, **Taiki Shishido**, **Yuki Koyo**, **Takehiro Yoshii**, **Reona Kawamura**, **Tsubasa Ishikawa**, **Yasuhiko Mizuguchi**, **Naoya Toyoshima**, **Hiroyuki Takamaru**, **Masayoshi Yamada**, **Masau Sekiguchi**, **Seiichiro Abe**, and **Yutaka Saito** contributed to data interpretation and critical revision of the manuscript. All authors approved the final manuscript.

## Funding

The authors have nothing to report.

## Conflicts of Interest

Seiichiro Abe is a Deputy Editor‐in‐Chief of DEN Open. The authors declare no conflicts of interest.

## Data Availability

Data sharing not applicable to this article as no datasets were generated or analysed during the current study.
